# Plant-based dietary biomarkers: from discovery to validation using mass spectrometry-based metabolomics

**DOI:** 10.3389/fnut.2026.1883491

**Published:** 2026-09-04

**Authors:** Gerben de Gier, Michiel G. J. Balvers, Edith J. M. Feskens, Renger F. Witkamp, Eva Jermendi

**Affiliations:** Division of Human Nutrition and Health, Wageningen University & Research, Wageningen, Netherlands

**Keywords:** dietary biomarkers, foodomics, mass spectrometry, metabolomics, plant-based foods

## Abstract

As diets shift globally toward plant-based foods, objective tools are needed to monitor intake and evaluate the resulting health outcomes. Biomarkers of dietary exposure offer complementary objective measurements for self-reported dietary assessment methods. Recent developments of mass spectrometry-based metabolomics have enabled the discovery of many emerging biomarkers for plant-based foods; yet a substantial gap remains between discovery and clinical application. This narrative review addresses the question: which plant-based food biomarkers are sufficiently validated for use in dietary exposure assessments, and which analytical and biological obstacles currently limit their application in population studies? Biomarkers reported in the literature were categorized as validated, emerging, or discovery-stage candidates. Analytical methodologies for biomarker quantification are also discussed, including high-resolution mass spectrometry platforms, and emerging techniques. Challenges identified in biomarker validation involve inter-individual metabolic variability, inadequate food specificity, and matrix effects in biological samples. Integration of foodomics databases with targeted validation studies potentially offers an efficient approach to speed up biomarker discovery. Multi-marker panels combining structurally different metabolites are promising for assessing complex plant-based dietary patterns.

## 1 Introduction

The global food system faces the challenge of ensuring human nutritional security while operating within planetary boundaries (1). One of the most effective strategies to address climate change and biodiversity loss while potentially improving human health is shifting the balance from animal-based toward more plant-based dietary patterns (2, 3). From a nutritional perspective, one key element of this shift is a protein transition: an intentional move from animal-based toward more plant-based protein sources. This transition is associated with reductions in greenhouse gas emissions, land use, and water consumption linked to Western dietary patterns (4). At the same time, a relative increase in plant-based foods may offer additional health benefits, depending on overall diet composition, for example when accompanied by higher intakes of dietary fiber and other plant-derived compounds with health-promoting properties. For example, the EAT-Lancet Commission’s Planetary Health Diet advocates sourcing proteins mostly from legumes and nuts; consequently it is estimated that this transition could decrease food related emissions by 17% while improving human health (4, 5). However, assessing and monitoring the potential nutritional and health consequences of such dietary shifts requires reliable, validated methods to measure the intake of plant-based foods (6, 7). Scientists currently rely on, among other things, food frequency questionnaires (FFQs) and 24-h dietary recalls to assess dietary intake and evaluate dietary patterns (6, 8). Recently, smartphone-based food diaries and image-assisted dietary assessment are helping to improve accuracy and reduce recall bias (7). These tools face limitations as they rely on self-reporting and memory, that are subject to bias and cannot capture bioavailability differences (9, 10). These challenges limit the objective monitoring plant-based dietary patterns, the evaluation of the health impacts, and the development of evidence-based dietary guidance. Therefore, alternative approaches are needed to improve the exposure assessment of plant-based foods.

Biomarkers of dietary exposure or also called biomarker of food intake (BFI) offer a complementary and objective approach to overcome the above-mentioned limitations, reflecting the intake or exposure to given foods or dietary patterns (11). Good biomarkers have specificity to the food or food group of interest, sensitivity to detect meaningful variations in intake, biological validity in reflecting actual consumption, and analytical feasibility for measurement in biospecimens (12). Biomarkers can range from specific food intake markers to complex metabolic signatures (11). While metabolomics methods enabled the discovery of numerous possible biomarkers, a substantial gap exists between biomarker identification and validation (13, 14). The Dietary Biomarkers Development Consortium (DBDC) outlined a complete three-phase approach to bridge the gap and identify, evaluate, and validate food biomarkers (15). The Food Biomarker Alliance (FoodBAll) developed a validation framework using eight criteria that provides a reliable standard for assessing dietary biomarker reliability (16–18). The validation framework is fundamental because many proposed biomarkers lack standardization in optimal biospecimen type, collection timing, storage conditions, and analytical methods (15, 19).

There are many challenges in biomarker validation for plant-based foods. Firstly, the high degree of inter-personal variability in biomarker concentrations due to gut microbiota differences, genetic differences in metabolic enzymes, or habitual dietary patterns can blur diet-exposure relationships (20, 21). Secondly, there is the issue of specificity as many of the plant-derived metabolites can be present in various food sources, making it difficult to assign biomarkers to specific foods (22). Thirdly, the timing of biomarker appearance, peak concentration, and clearance all remain inadequately characterized for many plant food biomarkers, limiting the understanding if they reflect recent intake or habitual consumption (18). Lastly, human biological samples such as plasma or urine are complex, which presents technical challenges for the development of sensitive, accurate, and precise analytical methods (23, 24).

To address these challenges, technological advances in analytical chemistry and data science are advancing biomarker discovery and validation (25). Ultra-high-performance liquid chromatography coupled with tandem mass spectrometry (UHPLC–MS/MS) is now a cornerstone method, offering high chromatographic resolution, sensitivity, and throughput compared to conventional high-performance liquid chromatography (HPLC) (26, 27). High-resolution mass spectrometers, particularly quadrupole time-of-flight (Q-TOF) and Orbitrap systems, enable accurate mass measurements with high resolution and precise metabolite identification (28). Complementary tools, such as foodMASST, FooDB, and the Human Metabolome Database (HMDB), are fast-tracking the annotation of dietary metabolites (29–32).

Despite the advances in biomarker discovery, the majority of identified compounds lack validation for individual plant foods. This gap limits the application of biomarkers for evaluating plant-based dietary interventions and providing personalized dietary guidance. Therefore this narrative review critically evaluates which biomarkers of plant-based food intake are sufficiently validated for dietary exposure assessment, and which analytical and biological barriers currently limit their use in population studies.

This review is framed around plant-based foods with emphasis to protein-rich legumes, pulses and nuts, however the scope extends to a broader set of plant-based food categories. Validated biomarkers for protein-rich plant foods remain few, and the better validated biomarkers available for foods such as citrus and whole grains serve as benchmarks, illustrating the validation principles applicable to other plant foods.

## 2 Literature selection strategy

The literature search was done in PubMed, Scopus and Google Scholar using the following search terms: (“dietary biomarker” OR “food intake biomarker” OR “biomarker of food intake”) AND (“plant-based” OR “legume” OR “whole grain” OR “metabolomics”) AND (“mass spectrometry” OR “LC–MS” OR “UHPLC–MS” AND/OR “targeted”). Supplementary terms included individual food names (soy, pea, lentil, bean, chickpea, oat, quinoa, citrus, apple, walnut, mushroom, carrot, tomato, green leafy vegetable, Brassica) combined with “biomarker” or “metabolomics.” The search was not specifically restricted by date, but the primary aim was on publications from 2000 to 2025, with foundational references from earlier years as well where it was necessary. Additional publications were identified by citation chasing, using one high-quality, relevant source to find additional related research. Inclusion criteria were: peer-reviewed primary research or review articles in English; studies reporting dietary biomarkers detected in human biospecimens (urine, plasma, or serum); and studies describing analytical methods relevant to plant-based food biomarker quantification. Exclusion criteria were: studies reporting exclusively animal data with no human evidence; studies focused on nutrient biomarkers (e.g., vitamins, minerals) rather than food-specific markers; and conference abstracts and gray literature. Biomarker classification was completed by applying the Food Biomarker Alliance (FoodBAll) validation framework and the Dietary Biomarkers Development Consortium (DBDC) three-phase criteria (12, 15, 18). While one of the motivations for this review is the protein transition, biomarkers from other plant food categories (e.g., citrus, whole grains) are included that illustrate validation principles applicable to other plant foods.

## 3 Biomarker validation framework

Dietary biomarker development and implementation require a systematic approach to ensure reliability, specificity, and applicability across diverse populations and research contexts (16, 18). Validated dietary biomarkers must meet a number of fundamental criteria to be considered reliable for assessing food intake (16, 18).

### 3.1 Criteria for biomarker validation

A consensus-based framework was developed by the FoodBAll, which proposes eight core criteria to ensure that BFIs provide precise measurements of food intake (16, 33).

**Plausibility** is the first standard criterion, meaning that a BFI is chemically correlated to and specific for the food or food group, with marginal occurrence in other food sources (16, 18). For instance, soy isoflavones have high plausibility, because they are mostly absent from other foods, making them evident biomarkers of soy intake (34, 35).

**Dose–response** means that biomarker concentrations change proportionally with the total amount of the food consumed (11, 18). This is essential for quantitative assessment, and it is well established for several BFIs, including some alkylresorcinols for rye and whole-grain wheat intake, furthermore for proline betaine for citrus consumption (16, 36).

**Time-response** evaluates the BFI kinetic profile after food consumption, including biomarker appearance, peak concentration, and clearance (18). Understanding these kinetic profiles is essential for verifying appropriate sampling windows. Some biomarkers reflect acute intake (detectable hours after consumption), while others indicate habitual intake (stable concentrations resulting from regular consumption) (16).

**Robustness** indicates that a BFI performs consistently when determined in a mixed meal, indicating that the signal is not considerably confounded by other co-ingested foods or by endogenic metabolic processes (16, 18, 22). Robustness needs to be confirmed in both controlled intervention studies and in observational settings of individuals.

**Reliability** requires comparison measurements with other objective biomarkers or with validated dietary measurements such as 24-h recalls or food records in free-living individuals, allowing assessment of whether biomarker levels reflect habitual dietary intake patterns (16, 18).

**Analytical performance** covers the technical and methodological requirements for accurate measurement, including detection limits and quantification (18, 26). MS-based methods are currently the analytical standard for dietary biomarker measurement given their superior selectivity (25–27). Matrix effects from co-occurring compounds in biological samples must also be assessed and controlled (24, 37).

**Stability** entails that the biomarker remains structurally intact under standardized conditions of sample collection, processing and storage (18).

**Reproducibility** means that measurements are comparable across the different analytical platforms, laboratories, and populations, crucial for cross-study comparisons (18).

### 3.2 Classification of biomarkers: validated, emerging, and discovery stage

Biomarkers have been classified into three categories based on their current level of validation status (Table 1) (16, 18). This acknowledges that biomarker development is an ongoing process, with compounds at various stages of evidence accumulation.

**Table 1:** Operational criteria for classifying BFIs as discovery-stage, emerging, or validated.

Criterion / evidence requirement	Discovery-stage	Emerging	Validated
Utility level	Level 3	Level 2	Level 1
Chemical identification	Tentative annotation based on accurate mass and/or MS/MS; confirmation with an authentic standard not required	Identity confirmed with an authentic reference standard	Identity must be confirmed with an authentic reference standard
Plausibility / food specificity	Chemically plausible; specificity to the food not yet assessed	Plausible and food-specific, alone or within a defined marker combination	Must be food-specific, giving a low risk of misclassification
Robustness (varied dietary background)	Not established	Demonstrated and marker stands out against a mixed diet in both intervention and observational settings	Confirmed in both controlled interventions and observational studies
Reliability / free-living validation	Not established	Not yet established, no comparison exists with another biomarker or dietary instrument	Required agreement with another objective biomarker and/or a dietary instrument shown in free-living individuals, ideally with time-matched sampling
Time–response (kinetics)	Kinetics typically uncharacterized	Characterized; time window for sampling estimated	Characterized; time window for sampling defined
Dose–response	Not yet evaluated	Demonstrated concentration increase with intake	The concentration measured in a human sample increases with the increased intake of the food.
Stability	Not established	BFI is appropriately stable during storage	BFI is appropriately stable during storage
Analytical performance	Discovery-grade, semi-quantitative (untargeted); no validated assay	Targeted method developed but not yet standardized across laboratories	Validated targeted quantitative method (selectivity, LOD/LOQ, accuracy, precision)
Reproducibility	Single laboratory	Not fully demonstrated between laboratories	Inter-laboratory reproducibility demonstrated.
Human studies	A controlled meal study or an observational study without replication	≥2 independent studies of contrasting design (≥1 controlled intervention AND ≥1 free-living / observational) confirming the marker	Discovery & independent confirmation of contrasting design & a reliability test in a free-living population. In practice ≥2–3 independent studies spanning controlled and free-living settings.

Classification follows the validation criteria of the FoodBAll framework and operationalized through the multi-level utility ranking of Cuparencu et al. (16, 18).

**Validated biomarkers** have substantial evidence supporting their reliability and utility for assessing dietary intake (33). These biomarkers meet several criteria: demonstrated dose–response relationships in controlled feeding studies, replication of findings in independent study populations, correlation with dietary intake in free-living populations, established analytical methods with documented performance characteristics, availability of reference standards for quantification, and understanding of major sources of variability (33). Examples include soy isoflavonoids (genistein, daidzein, and their metabolites) for soy intake (38), and alkylresorcinols and their metabolites DHPPA (3-(3,5-dihydroxyphenyl)-1-propanoic acid) and DHBA (3,5-dihydroxybenzoic acid) for wholegrain wheat and rye consumption (36). These biomarkers have been employed successfully in numerous epidemiological studies and intervention trials, with established protocols for measurement and interpretation (34, 36).

The validated classification does not imply perfection. Even well-validated biomarkers face limitations, such as inter-individual variability in metabolism, potential confounding from unexpected dietary sources, or technical challenges in measurement (39). However, the strengths and limitations of validated biomarkers are well-characterized, enabling informed use and appropriate interpretation. For validated biomarkers, the primary research needs involve refinement (improved analytical methods, better understanding of variability) rather than fundamental validation.

**Emerging biomarkers** show initial evidence but require additional validation before achieving widespread acceptance. These biomarkers have typically been identified through rigorous discovery-phase research and have demonstrated favorable characteristics in initial validation studies but lack the breadth of evidence supporting validated biomarkers (13). Specific gaps might include: validation limited to single populations or geographic regions, dose–response relationships established but not yet replicated, correlation with dietary intake demonstrated in controlled settings but not in free-living populations, analytical methods developed but not yet standardized across laboratories, or limited commercial availability of reference standards.

The pea biomarkers (2-isopropylmalic acid, asparaginyl valine, and N-carbamoyl-2-amino-2-(4-hydroxyphenyl) acetic acid) exemplify emerging biomarkers (40). These compounds have been identified through untargeted metabolomics, confirmed with authentic standards, shown dose-dependent responses to pea intake, and validated in an independent intervention study (41). However, their validation remains limited to European populations, their performance in free-living individuals consuming mixed diets requires further evaluation, and standardized analytical methods need broader dissemination.

**Discovery-stage biomarkers** have been identified through metabolomics studies but lack sufficient validation for confident interpretation (16). These candidates may have been detected as differentiating metabolites in a single discovery study, tentatively identified based on mass spectrometry data without confirmation using authentic standards, but not yet evaluated for specificity or dose–response. Discovery-stage biomarkers for lentils (hypaphorine, dopamine sulfate), chickpeas (protocatechuic acid), and white beans (pipecolic acid, methylcysteine) fall into this category (42). While these compounds show promise, substantial work remains before they can be considered reliable indicators of food intake.

The classifications are changing as evidence accumulates through additional studies, biomarkers advance from discovery to emerging to validated status. Some foods have received extensive investigation (particularly soy, due to its potential health effects), while others remain understudied despite nutritional importance (34). As the field of dietary biomarker research continues to advance, regular reassessment of biomarker classification ensures that newly validated biomarkers gain appropriate recognition.

## 4 Analytical methods

This section provides an overview of the analytical methodologies underpinning the discovery, validation, and application of BFIs. It integrates technological advances in metabolomics with key methodological considerations, including sample preparation, data processing, and sources of variability. Together, these elements are critical for the robustness and translational potential of biomarker-based dietary assessment.

### 4.1 Technology: untargeted and targeted metabolomics approaches

The identification and quantification of dietary exposure biomarkers for plant-based foods rely predominantly on metabolomics-based analytical approaches. These approaches can broadly be divided into untargeted metabolomics for biomarker discovery and targeted metabolomics for biomarker validation and quantitative assessment (43).

Untargeted metabolomics aims to detect as many metabolites as possible in a biological sample without prior selection of analytes. High-resolution mass spectrometry platforms such as ultra-performance liquid chromatography coupled to quadrupole time-of-flight mass spectrometry, Orbitrap-based systems, and nuclear magnetic resonance (NMR) spectroscopy are commonly used for this purpose (43). These technologies enable comprehensive metabolic profiling and have been widely applied in dietary intervention studies to identify candidate biomarkers of plant food intake, particularly for pulses and legumes. For example, urinary biomarkers of pea consumption, including 2-isopropylmalic acid and asparaginyl-valine, were initially identified using untargeted UPLC-QTOF-MS metabolomics (40). Moreover, the use of high-resolution MS enables confident structural elucidation, addressing a key analytical challenge relevant to biomarker research, namely isomeric interference. Such separation and confirmation strategies are directly transferable to biofluid analysis, where matrix effects and co-eluting metabolites may compromise specificity.

Despite these strengths, untargeted approaches are inherently semi-quantitative and often rely on tentative metabolite annotation. As such, their utility lies primarily in hypothesis generation and biomarker discovery rather than definitive quantification or validation.

Targeted metabolomics is therefore essential for the quantitative validation of candidate biomarkers. These approaches typically employ LC–MS/MS, often using triple quadrupole instruments operating in MRM mode. Targeted workflows provide high sensitivity, selectivity, and quantitative accuracy, particularly when supported by stable isotope-labeled internal standards. Recent developments have enabled multiplexed assays capable of simultaneously quantifying structurally diverse metabolites, including amino acid derivatives, polyphenols, and lipids, within a single run. However, targeted approaches remain constrained by their focus on predefined analytes and the availability of authentic reference standards.

Taken together, these approaches are complementary: untargeted metabolomics enables comprehensive biomarker discovery, whereas targeted metabolomics is required to achieve robust quantification and validation, highlighting the need for integrated analytical workflows.

### 4.2 Sample preparation of foods and biospecimen selection

Appropriate sample preparation and selection of biological matrices are critical for reliable biomarker detection and quantification of dietary biomarkers. These steps are essential to minimize matrix effects, improve analytical sensitivity, and assure accurate representation of metabolite concentration in complex biological samples. In the context of plant-based dietary biomarkers, this is particularly important due to the chemical diversity and often low abundance of diet-derived metabolites.

Urine and plasma are the most commonly used biofluids in dietary biomarker research, each reflecting different aspects of dietary exposure. Urine is particularly well suited for assessing short-term intake of plant-based foods, as many of these chemicals are rapidly absorbed, metabolized and excreted (44). Twenty-four-hour urine collections are generally considered the gold standard for exposure assessment, as they capture cumulative metabolite excretion over time and reduce variability associated with diurnal fluctuations (30). However, their use is limited by high participant burden and logistical constraints, particularly in large-scale or free-living studies. As a result, spot urine samples and postprandial collections are frequently employed in intervention studies to assess acute dietary responses, although these approaches require careful standardization and interpretation. In contrast, plasma may be more informative for compounds with longer half-lives or for lipid-soluble biomarkers.

Sample preparation strategies further influence biomarker detection and quantification. Common approaches include protein precipitation, enzymatic hydrolysis and solid-phase extraction to reduce matrix interference and improve sensitivity (45). The choice of preparation method depends on the chemical properties of the target metabolites and the analytical platform used. Inadequate or inconsistent sample preparation can introduce significant variability, underscoring the need for standardized protocols across studies.

### 4.3 Data processing, metabolite identification and annotation

Untargeted metabolomics generates large and complex datasets that require extensive computational processing and careful interpretation. Raw mass spectrometry data undergo multiple steps, including peak detection, alignment, normalization, and statistical analysis, to extract meaningful features from high-dimensional datasets (46). Multivariate statistical methods such as principal component analysis and partial least squares discriminant analysis are commonly applied to identify metabolites associated with specific dietary exposures (47, 48). However, while these approaches are effective for pattern recognition and candidate biomarker selection, they are sensitive to data preprocessing choices and may lead to overfitting or false-positive associations if not rigorously validated.

A critical bottleneck in untargeted metabolomics is the reliable identification and annotation of detected peaks. Initial metabolite annotations are typically based on accurate mass measurements and database matching using resources such as HMDB and FoodDB (17, 31). However, these assignments often remain putative, particularly for plant-derived metabolites that are structurally diverse and underrepresented in spectral libraries. Higher confidence identification requires confirmation through MS/MS fragmentation patterns.

In these metabolomics databases, a relatively small proportion of detected features reach the highest level of identification confidence. This limitation is particularly relevant in the context of plant-based dietary biomarkers, where many metabolites are shared across food sources or exist as structurally similar isomers, increasing the risk of misannotation. The limited availability of reference standards further constrains validation and quantitative translation. As a result, insufficient metabolite identification remains a major barrier to progressing from biomarker discovery to validated, application-ready markers, highlighting the need for improved spectral databases, standard availability, and harmonized annotation workflows.

### 4.4 Specificity: a central challenge in biomarker development

One of the central challenges in the development of biomarkers of plant-based dietary exposure is the limited specificity of many metabolites (18). In contrast to biomarkers derived from unique food components, many plant-derived metabolites originate from broad classes of phytochemicals that are widely distributed across multiple foods. Compounds such as polyphenol-derived metabolites, fatty acids, and certain amino acid derivatives can arise from diverse dietary sources, complicating the attribution of these metabolites to the intake of a single food item.

This limitation is particularly pronounced for plant-based diets, which are characterized by high chemical diversity and substantial overlap in metabolite composition between foods. For example, fatty acids such as α-linolenic acid and linoleic acid are present in a wide range of nuts, seeds, and vegetable oils, reducing their discriminatory power as intake biomarkers (49). Similarly, metabolites proposed as markers of specific legumes may also be present in other plant foods or arise from shared metabolic pathways, increasing the risk of misclassification (40). Importantly, this lack of specificity directly impacts the interpretability and applicability of biomarkers in epidemiological studies, where accurate differentiation between food sources is essential.

### 4.5 Variability and diversity

Variability in biomarker measurements represents a major limitation in the application of metabolomics for assessing plant-based dietary exposure (10, 11). This variability arises from a combination of biological, pre-analytical, and analytical factors, all of which can obscure true diet–biomarker relationships and reduce reproducibility across studies (14).

Biological variability is particularly pronounced for plant-based biomarkers and is driven by interindividual differences in metabolism, and habitual dietary patterns. These factors influence the absorption, biotransformation, and excretion of dietary metabolites, leading to substantial differences in biomarker concentrations even under controlled intake conditions (34, 50).

Pre-analytical variability further contributes to inconsistency. Factors such as biofluid type, timing of sample collection, fasting status, and storage conditions can significantly affect metabolite profiles (51, 52). This is especially relevant for plant-derived biomarkers, which often reflect short-term intake and exhibit rapid postprandial dynamics, making them sensitive to sampling conditions.

Analytical variability arises from differences in sample preparation, chromatographic separation, and mass spectrometric detection (53). The chemical diversity of plant-derived metabolites necessitates multiple analytical platforms (e.g., reversed-phase and HILIC chromatography), yet no single method provides comprehensive coverage (54). In addition, matrix effects and the limited availability of stable isotope-labeled internal standards can compromise quantification accuracy, particularly for emerging biomarkers. In untargeted workflows, variability in data processing and feature extraction further impacts reproducibility, as different algorithms and software tools may yield inconsistent results (55).

Collectively, these sources of variability complicate the interpretation, comparability, and generalizability of biomarker data. This poses a significant challenge for the translation of metabolomics findings into robust tools for dietary assessment. Addressing these limitations will require harmonization of sampling protocols, standardization of analytical workflows, and improved characterization of biological sources of variation (14).

### 4.6 Technical challenges and analytical developments

Analytical limitations remain a key constraint in the development and application of plant-based dietary biomarkers, despite substantial technological progress. One of the most critical challenges is the limited availability of authentic chemical and isotopically labeled standards for many metabolites identified in untargeted metabolomics studies (56). This restricts confident metabolite identification and hampers accurate quantification, particularly for structurally complex and low-abundance plant-derived compounds. As a result, many candidate biomarkers remain at the level of putative annotation, limiting their translation into validated tools for dietary assessment (43).

Recent advances in mass spectrometry–based analytical platforms have significantly improved both biomarker discovery and quantification. UHPLC–MS/MS remains the gold standard for targeted analysis, offering high sensitivity, selectivity, and throughput (57). The development of multi-analyte UHPLC–MS/MS methods now enables the simultaneous quantification of structurally diverse metabolites, including amino acid derivatives, polyphenols, and lipids, within a single analytical run (57). However, these approaches remain constrained by the requirement for predefined analytes and the availability of reference standards.

Complementary to targeted workflows, high-resolution mass spectrometry (HRMS), including Orbitrap and time-of-flight platforms, has transformed untargeted metabolomics by enabling comprehensive profiling of thousands of metabolites (57). These systems improve metabolite annotation through accurate mass measurements and MS/MS fragmentation data, facilitating the discovery of novel dietary signatures.

More recently, ion mobility spectrometry coupled to mass spectrometry (IM-MS) has emerged as an additional separation dimension based on molecular size and shape, enabling improved discrimination of structurally similar and isomeric metabolites (58). This is particularly important for plant-derived metabolites, which are characterized by substantial structural diversity and the presence of numerous isomeric compounds. Nevertheless, challenges in metabolite identification persist, particularly for isomeric compounds and metabolites not represented in existing spectral databases. As such, HRMS-driven discovery does not fully resolve the bottleneck of confident annotation.

Advances in chromatographic separation have further enhanced metabolome coverage. The complementary use of reversed phase and HILIC is now widely adopted to capture both lipophilic and polar metabolites (59, 60), which is essential for the chemically diverse metabolome associated with plant-based diets. Emerging techniques such as two-dimensional liquid chromatography (2DLC) and ultra-high-performance supercritical fluid chromatography (UHPSFC) offer additional separation power and improved resolution of structurally similar compounds (61, 62). However, these approaches increase analytical complexity and are not yet routinely implemented in large-scale studies.

Overall, the integration of high-resolution discovery platforms with targeted quantitative methods has led to hybrid workflows that are increasingly applied in dietary biomarker research. These combined strategies provide a robust framework for linking metabolite discovery to quantitative validation. However, despite these advances, significant challenges remain, including the need for harmonized analytical protocols. Addressing this gap will be essential to enable the reliable quantification and large-scale application of plant-based dietary biomarkers.

## 5 Biomarkers of plant-based foods

Dietary biomarker identification and validation follow a system from discovery to application, with different levels of evidence supporting their use in nutrition research. The 8 criteria for biomarker validation can be synthesized into a comprehensive four-level utility scale to rank them based on three validation criteria: plausibility, robustness, and reliability (Table 1) (16, 18). From the four utility levels, the first level represents validated biomarkers meeting all three aforementioned criteria, while levels 2 and 3 represent candidates needing additional validation, while level four specifies foods for which no biomarkers have been recognized (16, 33). Major metabolomics surveys have compiled hundreds of metabolites linked with plant food intake (63). Nonetheless, most emerging biomarkers are still at the discovery or early validation stages, which highlights the need for validation efforts.

### 5.1 Validated biomarkers

Validated biomarkers are the gold standard of dietary assessment, representing all criteria being plausibility, robustness, and reliability when compared with dietary intake measurements (16). For plant-based foods a small subset of biomarkers have achieved this validation level, primarily for soy, whole grains, and citrus fruits. Table 2 provides an overview of the biomarkers discussed in the following sections.

**Table 2:** Summary of validated food biomarkers of intake, organized by food source and validation status.

Food	Biomarker	Chemical class	Status	Detected in biofluid	Specificity & combination notes	References
Soy	*Genistein*	Isoflavone aglycone	Validated	Urine, Plasma	Food specific; combine with daidzein ± glycitein; stratify by equol-producer status	(16, 40, 50)
Soy	*Daidzein*	Isoflavone aglycone	Validated	Urine, Plasma	Core validated marker; trace in other legumes; use in multi-marker soy panel	(16, 40)
Soy	*Equol*	Isoflavone metabolite (microbial)	Validated	Urine, Plasma	Only 25–35% Western / 50–60% Asian populations produce equol; phenotype classification mandatory before use	(50, 64, 65)
Whole grain wheat/rye	*Alkylresorcinols (intact homologs)*	Phenolic lipid	Validated	Plasma	Specific to wheat, rye, barley; AR-C17:0/AR-C21:0 ratio differentiates wheat vs. rye	(36, 68)
Whole grain wheat/rye	*DHPPA*	AR urinary metabolite	Validated	Urine	Combine with DHBA; plasma t½: DHBA 10 h, DHPPA 16 h; 24-h urine recommended; UHPLC–MS/MS MRM preferred.	(67, 90)
Whole grain wheat/rye	*DHBA*	AR urinary metabolite	Validated	Urine	Combine with DHPPA; LC–MS/MS resolves matrix effects from co-eluting phenolics.	(67, 90)
Citrus (all types)	*Proline betaine (stachydrine)*	Quaternary ammonium betaine	Validated	Urine (preferred), Plasma	Citrus-specific at normal intake levels; rapidly excreted in urine, mostly cleared within ~14 h; combine with naringenin conjugates.	(71, 72)

AR, alkylresorcinol; DHBA, 3,5-dihydroxybenzoic acid; DHPPA, 3-(3,5-dihydroxyphenyl)-1-propanoic acid.

#### 5.1.1 Biomarkers for soy

Soybeans contain a distinct phytochemical profile dominated by isoflavonoids, that distinguishes soy from other plant sources (16, 40). Soy isoflavones are the most extensively validated dietary biomarkers, having evidence across controlled feeding trials, observational cohort studies, and multi-population validation (16). Food metabolomics have identified genistein, daidzein, and glycitein as the main isoflavone aglycones (40). Upon ingestion, soy isoflavones undergo considerable biotransformation. Intestinal microbiota β-glucosidases hydrolyze the glycoside conjugates to their corresponding aglycones (genistein, daidzein, glycitein), which are then absorbed or further metabolized (64). Daidzein undergoes consecutive reduction by gut bacteria and forms dihydrodaidzein then subsequently equol (65). There is a noticeable inter-individual variability in this biotransformation as only 25–30% of Western- and 50–60% of Asian populations have the necessary gut microbiota to produce equol, they are called “equol producers” (50). This distinction is important, and “equol producer” status should be classified before using equol as a biomarker of soy intake.

From an analytical perspective Saha et al. developed a rapid LC–MS method to quantify daidzein, genistein, and equol in human urine using a simple preparation procedure involving buffer and internal standard addition, followed by incubation and centrifugation (38). The method demonstrated a linear response with a limit of quantification (LOQ) of 3 ng/mL and is faster and less labor-intensive than earlier approaches. In comparison, Grace et al. described a more complex method including overnight incubation and solid phase extraction (SPE), followed by drying and reconstitution prior to LC–MS/MS analysis (66). Although more time-consuming, this method achieved a substantially lower LOQ, approximately 30 times lower than that reported by Saha et al. (38). This can be critical when analyte levels are low or near baseline. As a result, the method by Grace et al. may be more suitable for studies requiring high sensitivity, such as those involving low-dose exposure or subtle metabolic differences (66).

Regardless of achieving validation status among dietary biomarkers, some research gaps remain. Current biomarker panels rely heavily on equol in equol-producing populations. However, over half of the Western populations cannot produce equol, needing validated biomarker approaches for this phenotype (50, 65). Currently only about 15% of the validation studies explicitly classify results by “equol producer” status (33, 50).

Most validation studies are done using soybeans, tofu, and soy milk, which together represent almost half of soy consumption in Western markets. However ultra processed soy products, such as soy protein isolates or textured vegetable protein used in meat alternatives, and also fermented products, tempeh, miso, natto still lack biomarker validation. There are still knowledge gaps around the isoflavone bioavailability from protein isolates versus whole soybeans or the impact on isoflavone glycoside profiles of industrial processing, using heat, extraction methods or extrusion. Less than 5% of controlled feeding trials use processed soy (protein) ingredients as intervention foods (16).

The majority of controlled feeding trials test soy protein intakes of 20–60 g/day, equivalent to isoflavone amounts of 40–100 mg/day (35). However, the average soy intake in the Western populations is less than 10 g of soy protein/day which is comparable to 15–20 mg of isoflavones/day (34, 35). Validation at the typical intake levels is limited. Only 2 controlled feeding trials have tested lower soy intakes below 15 g protein/day (16). Therefore, future studies with more focus on testing 5–15 g soy protein/day would be suggested to confirm biomarker sensitivity at habitual intake levels.

Soy isoflavones satisfy every criterion of a validated biomarker, and they can be applied to assess soy intake in both intervention and observational settings.

#### 5.1.2 Biomarkers for whole grains and cereals

Whole grains contain phenolic lipids called alkylresorcinols (ARs), which are biomarkers for whole-grain wheat and rye intake (36). They are also present in barley, and triticale but are absent from other edible plants in notable concentrations. The metabolites 3-(3,5-dihydroxyphenyl)-1-propanoic acid (DHPPA) and 3,5-dihydroxybenzoic acid (DHBA) serve as urinary markers of wholegrain intake (67). ARs demonstrate several favorable characteristics as biomarkers: they are responsive to whole grain wheat and rye intake, correlate with various measures of consumption, and remain stable during food processing (67). The half-life of ARs in plasma is approximately 5 h, while metabolites have half-lives of 10–16 h in plasma and 10–12 h in urine (16). Specific ratios of AR homologs (AR-C17:0/AR-C21:0) can differentiate between wheat and rye consumption (68).

Early quantitative methods for AR metabolites, including DHBA and DHPPA, relied primarily on high-performance liquid chromatography (HPLC) coupled with coulometric electrode array detection, as described by Koskela et al. (69). Although these methods demonstrated acceptable recovery, linearity, and applicability for measuring whole-grain wheat and rye intake, they were limited by relatively low analytical specificity and restricted multiplexing capacity. In particular, electrochemical detection was vulnerable to interference from structurally similar endogenous phenolic compounds present in plasma and urine, which complicated accurate quantification in complex biological matrices. Furthermore, these approaches typically required relatively large sample volumes and extensive sample preparation, limiting throughput and scalability in large epidemiological studies.

The introduction of LC–MS/MS substantially improved the analytical performance of AR biomarker quantification. Modern methods combine protein precipitation with multiple reaction monitoring (MRM) on HILIC or reversed-phase columns, enabling sensitive and selective detection of DHBA, DHPPA, and intact AR homologs at low physiological concentrations (70). Because AR metabolites vary in polarity and ionization efficiency, optimized chromatographic separation and analyte-specific ionization conditions remain essential for accurate targeted analysis. Collectively, these advances have enabled sensitive and multiplexed profiling of AR metabolites in complex biological samples.

DHBA and DHPPA meet all validation criteria and are ready for application as markers of whole-grain wheat and rye intake, supported by validated LC–MS/MS assays. Their short elimination half-lives mean that timed urine collection should be regarded as a condition of their use.

#### 5.1.3 Biomarkers for citrus fruits

Citrus fruits have high concentrations of proline betaine (stachydrine), a quaternary ammonium compound that is an identifiable biomarker for citrus intake (71, 72). Proline betaine is also present in beans, spinach, and alfalfa, but the concentrations in them are much lower than in citrus, making it adequately specific for citrus intake assessment when consumed at normal levels (71, 73, 74).

Upon consumption, proline betaine is quickly absorbed in the small intestine without significant metabolism, appearing in plasma and urine primarily in its unmodified form (71).

Proline betaine, analytical evidence from LC–MS–based studies indicates that the compound is more robustly detected in urine than in plasma, although both matrices are feasible. Lloyd et al. (72) describe an analytical method where flow-injection ESI–MS enabled clear discrimination of dietary exposure groups based on urinary levels (72). More recent LC–MS metabolomics work has further confirmed the reliable detection of proline betaine and structurally related betaines in urine, while also highlighting matrix effects and the need for careful chromatographic separation in HILIC-based workflows (75). In contrast, although proline betaine is detectable in plasma, concentrations are typically lower and more variable due to rapid renal clearance, as already observed in comparative studies of blood and urine betaines (74). Consequently, LC–MS methods applied to urine generally provide higher sensitivity and better reproducibility for proline betaine, whereas plasma measurements are more suitable for short-term kinetic or postprandial studies (74).

Research gaps for citrus biomarkers include the inability to differentiate between citrus types (oranges, grapefruit, lemons, limes) using current biomarkers, effects of commercial juice processing on proline betaine content, and the limitation of proline betaine’s short half-life for studies with infrequent sampling.

Proline betaine is applicable as a biomarker of citrus intake, but due to its short half-life studies need to rely on frequent or timed sampling.

### 5.2 Emerging biomarkers

Emerging biomarkers demonstrate plausibility and robustness, detected in controlled and observational settings, but still lack adequate reliability validation in dietary assessment tools (16, 18). These biomarkers are candidates needing further validation studies to become validated biomarkers. Table 3 provides an overview of the discovery-stage biomarkers.

**Table 3:** Summary of emerging food biomarkers of intake, organized by food source and validation status.

Food	Biomarker	Chemical class	Status	Detected in biofluid	Specificity and combination notes	References
Peas	*2-Isopropylmalic acid*	Organic acid	Emerging	Urine	Most effective in combination with asparaginyl valine; also detected in wine (specificity concern); targeted LC–MS/MS assay in human biofluids still needed	(41, 76, 77)
Peas	*Asparaginyl valine*	Dipeptide	Emerging	Urine	Combine with 2-isopropylmalic acid; validated in European populations only; no free-living validation; no targeted biospecimen assay yet	(41)
Peas	*N-Carbamoyl-2-amino-2-(4-hydroxyphenyl) acetic acid*	Amino acid derivative	Emerging	Urine	Third marker in pea multi-marker panel; broader population validation needed	(41)
Apple	*Phloretin-2’-O-glucoside / phloretin metabolites*	Dihydrochalcone	Emerging	Urine, Plasma	Apple and Rosaceae-specific; variety and processing effects not fully studied; reliability vs. dietary instruments incomplete. Combine with arbutin to differentiate apples from pears.	(78–80)
Pear	*Arbutin + metabolites (hydroquinone sulfate/glucuronide)*	Phenolic glucoside	Emerging	Urine, Plasma	High concentrations in pears only among commonly consumed fruits; combine with phloretin for apple/pear differentiation.	(79, 81)
Chickpea	*Hypaphorine*	Alkaloid betaine	Emerging	Urine	Highest concentrations among common legumes; also present in lentils and beans; cross-legume controlled trials needed to establish specificity.	(42, 82, 83)
Whole grains / high-fiber foods	*Enterolactone, Enterodiol (lignans)*	Polyphenol-derived (microbial)	Emerging	Urine, Plasma	Shared across fiber-rich plant foods; microbiota-dependent; marker of dietary pattern rather than specific grain food	(84–87)

#### 5.2.1 Biomarkers for peas

Peas contain metabolite profiles distinct from other legumes, allowing specific BFI identification (16, 40, 41). The pea-specific candidate biomarkers that have been identified are 2-isopropylmalic acid, asparaginyl valine, and N-carbamoyl-2-amino-2-(4-hydroxyphenyl) acetic acid (41). These compounds were discovered through untargeted metabolomics and demonstrated differential time courses following pea consumption compared to control foods. All three markers increased in a dose-dependent manner and were confirmed in an independent intervention study (41). Importantly, 2-isopropylmalic acid and asparaginyl valine are indicated to be most effective when used in combination rather than as single markers (16). This multi-marker approach can address individual biomarker limitations and improve the overall classification, however, the limited commercial availability of pea biomarker reference standards hampers inter-laboratory standardization.

2-isopropylmalic acid as urinary biomarker was confirmed using MS/MS fragmentation patterns and authentic reference standards, elevating the confidence of annotation beyond putative identification (41). However, only relative amount were determined and no concentrations were determined. While biological plausibility and responsiveness to intake were established, further work is required to assess inter-individual variability, habitual intake associations, and long-term reproducibility.

A key limitation across the literature is the lack of fully validated targeted assays for 2-isopropylmalic acid in human biospecimens. The absence of absolute concentration data and standardized protocols currently constrains the translation of 2-isopropylmalic acid into observational studies. Future research should prioritize the development of targeted LC–MS/MS methods for urine and plasma, evaluation of intra- and inter-individual variability, and assessment of specificity relative to other legume and plant-based foods.

Complementary insights into the analytical measurement of 2-isopropylmalic acid are provided by studies conducted in complex food matrices, particularly wine, which indicates that this compound is not specific to a single dietary source. Ricciutelli et al. (76) focused on the identification and chromatographic separation of 2-isopropylmalic acid and its structural isomer 3-isopropylmalic acid using liquid chromatography coupled to ion trap and Q-Orbitrap mass spectrometry (76, 77). A validated a targeted UHPLC–triple quadrupole MS/MS method was also developed for the quantification of 2-isopropylmalic acid in wine (77). This study reported performance characteristics including linearity, repeatability, and recovery, demonstrating that sensitive and reproducible quantification of 2-isopropylmalic acid is achievable in chemically complex matrices. Although not conducted in human biospecimens, this work provides an important proof-of-concept for how targeted quantitative methods could be adapted for urine or plasma, a necessary step toward biomarker validation in large-scale nutritional epidemiology.

Targeted analytical methods for asparaginyl valine in human biospecimens are currently lacking, although untargeted LC–MS approaches have demonstrated its detectability and potential as a biomarker. While established LC–MS/MS workflows for dipeptides suggest that robust quantification is technically feasible, no validated, peer-reviewed assays are yet available. Similarly, for N-carbamoyl-2-amino-2-(4-hydroxyphenyl) acetic acid, quantitative analysis is achievable using LC–MS/MS or derivatization-based HPLC methods, but fully validated applications in human samples remain absent (16, 40, 41).

This highlights a clear research gap in the development and validation of targeted, quantitative methods for both compounds in biological matrices. Future work should focus on establishing standardized LC–MS/MS assays, including validation of sensitivity, specificity, and reproducibility, to enable their application in dietary biomarker studies.

Pea biomarkers are plausible and have been confirmed with authentic standards, but the absence of validated targeted assays and of absolute concentration data means they cannot yet be applied quantitatively. They can be suitable for confirming compliance in controlled interventions, where intake is known and sampling is time-matched, but not for estimating habitual pea intake in observational studies.

#### 5.2.2 Biomarkers for apples and pears

Apple peel and flesh contain phloretin-2’-O-glucoside, a dihydrochalcone distinctive for apples and closely related species (78, 79). Phloretin shows dose-dependent increases in controlled apple feeding trials and is detectable in everyday apple consumers, establishing plausibility and robustness (78, 80). However, formal reliability validation comparing phloretin levels with apple intake from FFQs and dietary records is incomplete. The assessment of phloretin level variability across apple varieties and processing methods, whole fruit, juice and sauce is still lacking. Pears contain arbutin (hydroquinone-β-D-glucopyranoside), which is only found in significant concentrations in pears and no other commonly consumed fruits (79).

Phloretin-derived metabolites are commonly measured in urine or plasma using targeted LC–MS/MS methods following sample preparation by solid-phase extraction or enzymatic deconjugation (79). Arbutin and its metabolites, including hydroquinone sulfate and glucuronide, are also primarily analyzed using LC–MS/MS approaches, often requiring optimized chromatographic conditions due to their high polarity and structural similarity to endogenous phenolic compounds (79, 81). The combination of phloretin- and arbutin metabolites enables distinction of apple from pear consumption, showing the value of combined markers also for closely related foods.

Phloretin and its conjugates are the emerging biomarkers for apple intake and arbutin for pear intake, and the two should be measured together, since neither distinguishes apple from pear reliably on its own. Both are suitable for compliance assessment in controlled interventions, however free-living validation is still required before they can be used to estimate habitual intake.

#### 5.2.3 Biomarkers for chickpeas

Chickpeas contain hypaphorine (N, N, N-trimethyltryptophan betaine), a quaternary ammonium compound (42, 82). While hypaphorine is also found in lentils and some beans, and other legumes, chickpeas are the most studied as a dietary source in BFI validation context (42, 82). Hypaphorine is rapidly absorbed and excreted mainly unchanged through urine, while small amounts undergo demethylation and conjugation (83). In the Mediterranean diet intervention study by Agongo et al. (82), hypaphorine served as a biomarker for chickpea/hummus consumption, demonstrating significant increases during intervention periods rich in legumes (82). This demonstrates the biomarker’s utility for compliance monitoring in dietary interventions. As lentils and beans also contain hypaphorine, controlled trials comparing biomarker response across legume types are still needed to establish chickpea specificity.

As hypaphorine also occurs in lentils and other beans, it cannot assess intake to chickpeas specifically in a mixed diet. Its appropriate current use is to confirm legume intake within a controlled intervention, or to contribute to a multi-marker legume panel.

#### 5.2.4 Biomarkers for whole grains and fiber rich foods

Lignans, including enterolactone, enterodiol, matairesinol, and secoisolariciresinol, serve as biomarkers for whole grains and fiber-containing plant foods (84). Nevertheless, their classification needs explanation, because lignans are more emerging rather than validated biomarkers, as they are present in multiple plant foods and they need substantial gut microbiota conversion to reach their measurable forms (enterolactone and enterodiol). Lignan excretion is associated with dietary fiber intake, and high fiber diets correlate with higher enterolactone levels, which steered them to markers of high fiber dietary patterns more broadly than of specific grain foods (85).

A method for the quantification of lignans in food using GC–MS has been described by Peñalvo et al. (86). This method was validated for six dietary lignans with a sample pretreatment includes hydrolysis, derivatization and SPE to concentrate. Later work by Nørskov et al. describes quantification of lignans in both urine and plasma using a LC–MS approach (87). Here, hydrolysis and SPE cleanup was performed. The use of LC–MS here leads to much shorter injection time (less then 5 min) compared to the mentioned GC–MS approach (approximately 30 min) making it much more suitable for high-throughput analysis. Moreover a much lower limit of quantification was achieved using LC–MS (0.024 ng/mL) for most of the lignans.

Whole grain oats contain avenanthramides found in significant concentrations only in oats among other commonly consumed cereals (16, 88). This uniqueness makes avenanthramides solid candidates being oat-specific biomarkers. Following oat consumption, they can be detected in urine and plasma, demonstrating plausibility (88). However, validation data, including dose–response studies, confirmation in free-living individuals, and the robustness against a mixed dietary background is still limited. Avenanthramides are priority targets for a controlled oat feeding trial with biofluid collection. They can be quantified in urine and plasma using targeted UHPLC–MS/MS using reversed-phase chromatography (33).

Quinoa is a popular plant protein source and contains alkylresorcinols with a distinctive homolog pattern, that are AR-C18:0 and AR-C22:0 (33). They differ from the alkylresorcinol profile of wheat and rye (89). This signature offers potential for differentiating quinoa consumption from other cereal grain intake using LC–MS/MS developed for wheat and rye profiling (90). Therefore, quinoa represents an early-stage candidate biomarker and would be recommended to prioritize in future biomarker research.

Lignans reflect intake of a broad range of fiber-rich plant foods rather than any single food, and depend on gut microbial conversion, so they are better considered as markers of a dietary pattern than of a specific food. Avenanthramides are oat-specific, which makes them a possible candidate in this group, but they lack dose–response data, free-living confirmation, and demonstrated robustness against a mixed dietary background. The quinoa alkylresorcinol signature is chemically distinctive and analytically tractable, and is therefore recommended as a priority target for future validation.

### 5.3 Discovery-stage biomarkers

Discovery-stage biomarkers demonstrate chemical plausibility, they are chemically correlated with specific foods but have only been reported either in controlled interventions or observational cohorts, thus lacking the robustness and reliability of validation required for standard applications (16). Table 4 provides an overview of the discovery-stage biomarkers discussed in the following sections.

**Table 4:** Summary of discovery-stage food biomarkers of intake, organized by food source and validation status.

Food	Biomarker	Chemical class	Status	Detected in biofluid	Specificity and combination notes	References
Lentil	*Hypaphorine*	Alkaloid betaine	Discovery	Urine	Moderate concentrations; not specific vs. chickpeas; no free-living validation; additional lentil-specific markers needed.	(42)
Lentil	*Dopamine sulfate*	Amino acid derivative	Discovery	Urine	Also identified as banana biomarker; insufficient food-level specificity; cannot be used alone for lentil attribution.	(91)
Lentil	*Flavan-3-ol-derived metabolites (DHPVL conjugates)*	Polyphenol-derived	Discovery	Urine	Shared across legumes and plant foods; specificity for lentils not established; may support a broad legume panel.	(16)
White/dry beans	*Hydroxyjasmonic acid*	Oxylipin (jasmonate)	Discovery	Urine	Detected in one controlled feeding trial only; no replication; present in other plant foods.	(42, 95)
Dry beans	*Pipecolic acid*	Amino acid	Discovery	Serum/Plasma	LC–MS/MS quantification exists (HILIC, 0.05–50 μmol/L); specificity vs. other legumes not established.	(42, 93)
Dry beans	*S-Methylcysteine*	Amino acid derivative	Discovery	Urine, Plasma	Quantification validated in biofluids; food specificity in mixed-diet settings not confirmed; combine with pipecolic acid.	(42, 94)
Walnuts	*Urolithins A, B + conjugates*	Polyphenol-derived (ellagitannin, microbial)	Discovery	Urine	Also produced from pomegranate and other ellagitannin sources; entirely microbiota-dependent; multi-urolithin isomer panel recommended.	(96–99)
Brazil nuts	*Selenium species*	Mineral/elemental	Discovery	Urine, Plasma	Present in seafood, meat, and cereals; not nut-specific; requires ICP-MS rather than standard LC–MS/MS; use as supporting marker only with dietary context.	(100, 101)

#### 5.3.1 Biomarkers for lentils and white beans

Lentils also contain hypaphorine at moderate concentrations (42). Other biomarker candidates are dopamine sulfate, and flavan-3-ol derived metabolites, such as dihydroxyphenyl-γ-valerolactone (DHPVL) conjugates, but their specificity for lentils versus other legumes remains unclear (16). Moreover, dopamine sulfate is particularly challenging, as it was recently found to be a biomarker predicting banana intake, demonstrating how discovery-stage biomarkers can fail the criterion for plausibility (91). This highlights the value of cross-food specificity testing before considering candidate BFIs.

White beans and dry beans contain the candidate biomarkers hydroxyjasmonic acid, pipecolic acid, and S-methylcysteine, though the concentrations and specificity of the data are limited (16, 42). Bean biomarkers are one of the most significant gaps in plant-based food biomarker validation, given the nutritional importance of common beans to plant-based diets.

The presence of multiple biomarkers in lentils creates a need for their simultaneous measurement in a single analytical run. Simultaneous quantification of structurally diverse metabolites such as alkaloids, amino acid derivatives, and polyphenol derived metabolites in one analytical workflow presents significant analytical challenges but is increasingly feasible with modern LC–MS/MS instruments. Hypaphorine has historically been quantified using fluorometric and chromatographic methods in complex food and biological matrices (83). Concurrently, targeted LC–MS/MS assays have been successfully deployed to measure intact sulfate and glucuronide conjugates of dopamine and other catecholamines directly from biological samples without hydrolysis, highlighting the ability of optimized LC–MS methods to resolve and quantify biotransformation phase II metabolites in a single run (92). Taken together, these advances support the recommendation of developing a targeted, multi-analyte LC–MS/MS method with careful chromatographic separation, appropriate internal standards, and validated calibration curves to quantify hypaphorine, dopamine sulfate conjugates, epicatechin metabolites, and other flavan-3-ol-derived metabolites simultaneously with high specificity and quantitative accuracy.

Simultaneous quantification of all white bean–derived metabolites necessitates analysis in both urine and serum, as these biomarkers are distributed across these two biofluids. For amino acid–related analytes like pipecolic acid, validated LC–MS/MS methods employing HILIC or reverse-phase separation without derivatization have demonstrated good linearity, recovery, and low limits of quantification. For example, pipecolic acid has been quantified in plasma over a wide linear range (0.05–50 μmol/L) with a simple protein precipitation and HILIC-MS/MS approach, achieving high recovery and low LOD/LOQ values (93). Similarly, S-methylcysteine (a methylated cysteine derivative) has been quantified using targeted LC–MS/MS in biological matrices, demonstrating acceptable linearity and precision for both urine and plasma samples, making this a practical strategy for polar amino acid derivatives (94). For jasmonate-related phytohormones, including hydroxyjasmonic acids and their reduced forms such as hydroxydihydrojasmonic acids, UPLC–MS/MS methods with solid-phase extraction provide sensitive and precise quantification (95). Collectively, these strategies support development of a targeted LC–MS/MS workflow capable of simultaneously quantifying pipecolic acid, methylcysteine, hydroxyjasmonic acid, and hydroxydihydrojasmonic acid with high sensitivity, selectivity, and reproducibility in complex biological or plant matrices. However, a gap remains in the lack of a fully validated platform capable of simultaneously quantifying both amino acid-derived and jasmonate-related metabolites across serum and urine within a single harmonized workflow.

No marker for lentils or beans is currently usable as BFI. Hypaphorine is also present chickpeas, while pipecolic acid and S-methylcysteine occur across other legumes. Until a controlled cross-legume specificity trial has been completed, these compounds should be reported as general legume biomarkers.

#### 5.3.2 Biomarkers for nuts

Most proposed biomarkers for nut intake do not have sufficient level of specificity as individual biomarkers, reflecting high chemical overlap between nuts and other plant foods. In all nuts, seeds, and vegetable oils, fatty acids such as α-linolenic acid are present, limiting their function (49). Urolithins cannot distinguish walnut intake from pomegranate or other ellagitannin-containing foods, and are therefore not food-specific biomarkers (96). Detection in urine within a few hours of walnut consumption provides preliminary time-response characterization, in addition multi-urolithin panels (urolithin A, B, and glucuronides) improve their specificity compared to a single metabolite measurement (97, 98). On the other hand, urolithin production is gut microbiota-dependent, generating inter-individual variability complicating quantitative applications (96, 99).

Brazil nuts are associated with selenium intake, and HPLC-ICP-MS methods offer sensitive and selective quantification of selenium in both urine and plasma (100). However, selenium is present in many food categories including seafood, meat, and cereals, limiting it being a Brazil nut-specific biomarker without precise dietary information (101). Multi-metabolite methods combining a number of nut-associated compounds are recommended for improving specificity. Detection of both urolithins and selenium in urine and other biological substances has already been established by optimized LC–MS methods (49). For urolithins, this method employs reverse-phase LC with a methanol water gradient and has been extended to include multiple urolithin isomers even when authentic standards are limited, aided by relative response factor determination (99). In contrast, selenium quantification often relies on elemental mass spectrometry approaches such as inductively coupled plasma mass spectrometry (ICP-MS) with chromatographic separation (HPLC-ICP-MS) (100). For simultaneous profiling in the same sample, a hyphenated workflow could combine conventional LC–MS/MS for organic analytes like urolithins with post-column split to an ICP-MS detector or parallel analysis streams, ensuring that organic metabolites and elemental selenium species are both quantified with minimal compromise (100).

Nut biomarkers are currently too non-specific to be useful for attributing intake to a particular nut. Urolithins cannot distinguish walnuts from other ellagitannin-containing foods, and selenium also reflects intake from seafood, meat, and cereals as well as Brazil nuts.

#### 5.3.3 Biomarkers for green leafy, bulb, and stem vegetables

Green leafy, bulb, and stem vegetables, including spinach, lettuce, endive, asparagus, artichoke, and celery are representing a category for which some candidate BFIs currently exist. The most commonly reported post-consumption metabolites such as carotenoids, vitamin K1, nitrate, and oxalate were excluded for lack of food specificity, as were phenolic acids including p-coumaric acid and caffeic acid detected after lettuce consumption, and kaempferol glucosides identified after endive intake, all of which are widely distributed across unrelated food sources (102). The most plausible dietary biomarkers identified based on food composition data are spinach-specific flavonoid conjugates, glucuronide and sulfate derivatives of spinacetin and TMM (5,3′,4′-trihydroxy-3-methoxy-6,7-methylenedioxyflavone), which have high specificity for spinach according to multiple food databases and were detected in plasma after ingestion of ^13^C-labeled spinach (102, 103). However, they have not been measured in urine, no dose–response relationship has been established, and no controlled feeding trial has been designed with their identification as a primary objective. For asparagus, volatile sulfur metabolites of asparagusic acid are detectable in urine by GC–MS and are chemically specific, but marked inter-individual variation in their production and analytical instability limit their utility as BFIs (102, 104). Eventually no plausible candidates have been identified for artichoke, celery, lettuce, endive, or rocket. A combination of more metabolites using untargeted analyses, instead of the targeted single biomarker analysis is a favorable approach to find more specific and robust BFIs for green leafy, bulb, and stem vegetables (102).

This food group remains the least well characterized, with no biomarker yet meeting the specific food attribution. The most frequently reported metabolites, including carotenoids, vitamin K1, nitrate, and oxalate, are distributed too widely across plant foods, and no plausible candidate has so far been identified for artichoke, celery, lettuce, endive, or rocket. Biomarker discovery for these vegetables should therefore be regarded as a priority.

### 5.4 Proposed targets for future biomarker development

Based on the metabolomics literature and the food composition data available in databases such as FooDB and HMDB, the following targets have been identified for future biomarker development. These foods have been selected based on their nutritional importance to plant-based diets, their chemical distinctiveness, or the availability of compelling preliminary evidence for specific candidate compounds. Table 5 provides an overview of the proposed new biomarkers.

**Table 5:** Summary of priority targets of biomarkers of intake, ordered by strength of existing human evidence.

Food	Candidate biomarker	Detected in biofluid	Key limitation	References
Common beans	*Pipecolic acid, S-methylcysteine*	Serum	Shared with other legumes; cross-legume specificity unestablished.	(93, 94, 105)
Faba beans	*Divicine, isouramil and conjugates*	Urine, plasma	No human biofluid PK data; safety in G6PD-deficient populations.	(106, 107)
Mushrooms	*Ergothioneine*	Urine, plasma	Present in other food sources, uptake varies with SLC22A4 genotype.	(108, 110, 111)
Tomatoes	*Esculeogenin B sulfonate; hydroxy-esculeogenin B isomers*	Urine	Putative identification only; not validated for processed tomato products.	(112, 113)
Brassica vegetables	*3,3’-Diindolylmethane (DIM)*	Urine	Specific to glucobrassicin-rich species; not pan-Brassica.	(114)
Carrots	*Falcarinol*	Serum	Very short t½ requires postprandial sampling; shared with other *Apiaceae*.	(115, 116)

Common beans are a central to plant-based diets yet remain understudied. Promising, but not yet food specific candidates are serum pipecolic acid and S-methylcysteine, identified in a controlled human feeding trial (*n* = 46, 250 g/day) and subsequently confirmed in an independent observational cohort of 212 participants from the Polyp Prevention Trial (105). The compounds showed dose–response associations with self-reported dry bean intake (r > 0.95). However their primary limitation is specificity, as both occur in other legumes and have been discussed separately as candidates for lentils and white beans (Section 5.3.1). The research priority is therefore a controlled cross-legume specificity trial comparing common beans, lentils, chickpeas, and peas in the same participants, using validated targeted LC–MS/MS methods already available for both compounds (93, 94).

Faba beans have a rapidly growing market and contain a phytochemically unique profile. The pyrimidine glucosides vicine and convicine are virtually absent from other commonly consumed foods, making them plausible BFI candidates. Upon ingestion, they are hydrolysed to the redox-active aglycones divicine and isouramil, which are detectable candidate urinary analytes. A validated high-throughput LC–MS/MS method for vicine and convicine in faba bean flour exists (106) and controlled human feeding studies with metabolite profiling of plasma and urine have established technical feasibility (107). No human pharmacokinetic or BFI-specific study has been conducted. Development of a targeted biofluid assay and a controlled single-dose human study with defined vicine/convicine intake would be a clear and achievable next step.

Mushrooms are not botanically plants, but they are staple of plant-based eating patterns and are widely used as meat substitutes. Ergothioneine, a sulfur-containing histidine derivative is a biomarker candidate in mushrooms (108). Uniquely among biomarkers, it is strongly retained rather than rapidly excreted, giving a long sampling window that could reflect habitual rather than recent intake (109). Bioavailability from *Agaricus bisporus* has been demonstrated in a controlled human dose–response study, in which ergothioneine rose postprandially following gradual mushroom doses (110). Yet, food-level specificity is unproven because it also occurs in fermented foods, therefore a controlled trial contrasting mushroom intake against other food sources is required. Ergothioneine uptake also depends on genetic polymorphism in SLC22A4 gene that produces inter-individual variation (111). This is analogous to the equol-producer problem in soy, which means that transporter genotype may need to be characterized, or the marker restricted to qualitative classification, until its relevance is quantified.

Tomatoes are among the most globally consumed vegetables. Two independent LC-HRMS metabolomics studies identified esculeogenin B sulfonate and hydroxy-esculeogenin B isomers, phase II metabolites of the steroidal glycoalkaloid esculeoside B, in human urine after tomato juice consumption (112). All identifications remain putative, as they have been only confirmed by MS/MS fragmentation but not by authentic reference standards. A critical limitation is that esculeoside B concentrations are substantially altered by heat and acid processing (113), raising uncertainty about the applicability of these biomarkers to ketchup, tomato paste, and canned products, which constitute a large fraction of global tomato consumption. Reference standard acquisition and dose–response validation across both fresh and processed tomato products are the essential next steps.

Brassica vegetables, such as cabbage, Brussels sprouts, kale, and cauliflower are globally consumed and important vegetables yet are systematically overlooked relative to broccoli. Unlike broccoli, dominated by glucoraphanin and sulforaphane, these other brassicas are the principal dietary sources of glucobrassicin (producing indole-3-carbinol, I3C) and sinigrin (producing allyl isothiocyanate, AITC). Urinary 3,3′-diindolylmethane (DIM), the principal condensation product of I3C formed in the stomach, has been validated as a biomarker of glucobrassicin intake in a randomized crossover trial (*n* = 25) using a validated LC-ESI-MS/MS method with deuterated internal standard (114). Urinary DIM was consistently and significantly higher after Brussels sprouts than after cabbage feeding, and differences in glucobrassicin exposure across Brassica species were reliably reflected in urinary DIM levels. This makes DIM the most analytically advanced Brassica BFI candidate outside of broccoli, and the immediate priority is robustness evaluation in free-living populations consuming mixed Brassica-containing diets.

Carrots are the fifth most consumed vegetable globally and contain polyacetylenic oxylipins, primarily falcarinol and falcarindiol (115), that are almost exclusively found in the Apiaceae family (carrot, parsnip, celery, fennel), with carrot being the dominant dietary source. A validated LC–MS/MS method for falcarinol in human serum has been developed and applied in 18 healthy volunteers following carrot consumption: serum concentrations peaked at 1 h post-intake and declined with a plasma half-life of approximately 1.5 h (116). This represents the only existing human pharmacokinetic dataset for a carrot-derived compound using a validated targeted method, and it establishes both plausibility and a preliminary time-response profile (116). The very short half-life necessitates postprandial rather than fasting sampling, which is the principal analytical constraint. A controlled dose–response study with standardized carrot products is the logical next step, building directly on this existing methodological foundation.

## 6 Discussion

Recent advances in untargeted metabolomics have accelerated the discovery of plant-based biomarkers, nevertheless only a limited number of candidate biomarkers have progressed to fully validated biomarker status (18). Many compounds demonstrate associations with specific foods, but lack validation in intervention trials, dose–response studies, and replication by various labs that would be required to advance beyond the discovery stage (18). Upcoming studies should focus not only on identifying novel biomarkers, but also on systematic conducting dose–response experiments, confirming reproducibility across labs, and verifying robustness in free-living populations eating mixed diets. The limitations that explain the gap between discovery and application are the limited availability of reference standards essential for absolute quantification, the lack of time-matched sampling in most trials, and the insufficient characterization of inter-individual variability (15, 18).

Compounds such as soy isoflavones and proline betaine demonstrate relatively high specificity, however these examples remain exceptions while specificity for most biomarkers remains limited (16, 35). As plant-based diets are chemically diverse and often contain combinations of overlapping food sources, relying on single biomarkers may lead to misclassification of dietary intake. This limitation underlines the need for more sophisticated approaches and food-specific metabolite signatures, such as considering multi-marker approaches. This strategy is advocated as one of the most implying solutions to the specificity problem, but the field is yet divided on the reliability of these panels for epidemiological application (16, 33). Proponents argue that the advantages of multi-marker strategies over single-metabolite measurements would justify their use even before the individual biomarkers would be fully validated, and that the use of validated single-metabolite biomarkers might never be achievable for most of plant-based foods given the large chemical overlap between them (20, 48). Critics say that classification models are trained and tested within populations with similar dietary patterns, creating overfitting risks, creating more limitations in diverse populations. Large-scale external validation studies across diverse populations should be systematically performed, before using multi-marker panels routinely (33, 63). This is an important controversy in the biomarker field, and both perspectives are valid depending on the research question and the degree of validation evidence available.

Combining several markers into a panel compensates for the limited specificity of individual biomarkers, improving the classification of consumers vs. non-consumers of specific foods. Because plant-based dietary patterns consist of complex combinations of foods, exposure results in overlapping metabolite profiles rather than isolated biomarker responses (11, 20). In addition, multi-marker approaches may partially compensate for inter-individual variability caused by differences in gut microbiota composition, metabolism, and habitual dietary intake (16). Recent studies have shown the combination of different biomarkers in a single assay (48, 63). However, there is still a lack of standardized analytical and statistical workflows (23, 55). Upcoming research should therefore focus on developing and validating reproducible multi-marker methods and forming consensus on the interpretation of complex metabolite signatures.

Despite major technological advances in mass spectrometry-based metabolomics, there are important analytical limitations (23, 28). A key issue is the limited availability or high cost of authentic and isotopically labeled standards for many plant-derived metabolites, restricting identification, accurate quantification and inter-laboratory comparability. Without reference standards, validated biomarker status cannot be achieved. To solve this issue, coordinated investment is required, ideally organized through official consortia such as the Dietary Biomarkers Development Consortium (DBDC) (15). In addition, matrix effects, inconsistent sample preparation, and differences between analytical platforms contribute to variability and reduce reproducibility across studies (23, 27). Although the advanced MS-based techniques have substantially improved in analytical performance, harmonized protocols and standardized workflows are still lacking. Addressing these workflows may benefit the translation of metabolomics discoveries into robust biomarker analysis. The alkylresorcinol biomarkers for which both laboratory comparisons among multiple labs and standardized protocols have been developed provides a useful template that other plant food biomarker research groups could adopt (70, 90).

A major bottleneck in untargeted metabolomics for dietary biomarker discovery is the accurate annotation and identification of detected metabolic features. Although high-resolution mass spectrometry platforms can detect thousands of compounds simultaneously, only a relatively small proportion of these signals can be confidently assigned to known metabolites (28, 63). Moreover, the fact that data processing tools, such as MZmine, XCMS, and MS-DIAL, might yield inconsistent results depending on algorithm versions and parameter choices compounds the problem (46, 55). This challenge is pronounced in plant-based biomarker research because plant metabolites are structurally diverse and often occur as isomeric compounds with nearly identical mass spectra. In addition, many food-derived metabolites and their biotransformation products are still underrepresented in existing spectral libraries and metabolomics databases, such as The Human Metabolome Database and FooDB (31, 32). As a result, candidate biomarkers can remain unidentified or can be incorrectly annotated and therefore limiting the progression of biomarker validation. A major research gap therefore remains in the development of harmonized annotation workflows that enable confident comparison and metabolite validation across labs.

Inter-individual variability represents a major challenge in the interpretation of plant-based dietary biomarkers. The absorption, metabolism, and excretion of plant-derived compounds are strongly influenced by the host’s gut microbiota composition (16). For example, the conversion of daidzein into equol for soy intake does not occur in all individuals (64, 65). Such biological differences complicate the interpretation of results and comparison across populations. Regarding microbiota-dependent metabolites there is an ongoing controversy, for example if equol and also urolithins should be classified as food intake biomarkers or as biomarkers for gut microbial phenotypes (64). On one hand, they depend on gut microbial conversion which makes them unreliable as quantitative biomarkers, therefore their use should be limited to qualitative classification, such as equol-producer vs. non-producer status, instead of continuous indicators of soy or walnut intake (50, 64, 99). On the other hand, with adequate sample sizes and known gut microbiota characteristics, these biomarkers can be still useful when individual variability averages out to reflect food-level exposure patterns (33). The appropriate application of these gut microbiota-dependent biomarkers likely depends on the research question, study design, and the possibility of microbiota characterization in the study population. Biomarker research should therefore increasingly integrate microbiome characterization and personalized metabolic profiling to improve interpretation and applicability.

A structural limitation in the present plant-based biomarker literature is the disconnect between the timing of biological sample collection and registration of food intake. Most studies collect blood/urine samples and dietary assessments independently, often with considerable time between them, making it challenging to assess the reliability of short elimination half-life dietary biomarkers (18). Self-reported diets, with a time delay, are prone to bias. Time-matched sampling, such as the collection of biospecimens during a recorded period of food intake, is essential for reliability validation in free-living populations. This criterion is already well established in the biomarker validation framework (16, 18), yet it remains the exception in current research. Prospective studies that incorporate time-matched sampling aligned with the elimination kinetics of biomarkers would greatly speed up the progression of candidate biomarkers from discovery to validated status.

Another missing piece of research is the lack of biomarker validation for processed plant-based protein products. Most controlled feeding trials underpinning recent plant-based biomarker evidence use whole plants. Yet the dominant protein sources on the market are ultra processed products, e.g., soy protein isolates, pea protein isolates, textured soy protein, mycoprotein, and some fermented products. Industrial processing using heat treatment, extrusion, extraction, and fermentation can alter phytochemical profiles, glycosides, and even bioavailability, resulting that validated whole plant food biomarkers might not be equivalent for their ultra processed forms. Hardly any trials have used processed plant-based proteins for their intervention (16), representing a research gap given that these products are the primary means of plant protein intake. Future validation studies should include processed plant proteins alongside their whole-plant equivalents (117).

The development of validated biomarkers for plant-based foods has important implications for nutritional research and the transition toward more sustainable dietary patterns. Objective biomarkers of dietary exposure could substantially improve the monitoring of dietary intake in both intervention studies and free-living populations (11, 14). This is especially relevant in the context of the ongoing protein transition, where accurate assessment of plant-based dietary intake is essential for evaluating health and environmental impact (1, 118).

Several developments will likely transform the plant-based dietary biomarker landscape. The expansion of foodomics spectral databases, mostly The Human Metabolome Database and FooDB in combination with the cross-study spectral searching tool foodMASST, will likely reduce the annotation strain and fast-track the plant-based biomarker identification (29, 31). When these databases will integrate industrial processing-related metabolite differences, they will strengthen biomarker development for processed plant-based protein products that are currently lacking validated biomarkers.

Minimally invasive sampling, such as finger-prick technologies, hold promise for expanding sampling frequency in large-scale and longitudinal studies reducing excessive participant burden (16). Frequent sampling would be important for plant-based biomarkers with short elimination half-lives for sufficient coverage of habitual dietary intake in epidemiological settings (16, 18).

Ultimately, the integration of dietary biomarkers with metabolomics could further improve the understanding of metabolic responses to plant-based diets (14). However, before these applications can be fully realized, substantial efforts are still required to standardize analytical methods, validate biomarkers across diverse populations, and improve the interpretation of complex metabolomic signatures (18, 33, 63).

## 7 Conclusion

Dietary biomarker research for plant-based foods has advanced considerably in recent years, driven by improvements in mass spectrometry-based metabolomics and analytical chemistry. However, a clear hierarchy of validation status emerges as for the majority of plant foods, importantly for legumes, essential to the protein transition validated BFIs do not yet exist. The inadequate availability of reference standards for plant metabolites is one of the most actionable technical barriers, as it prevents absolute quantification and inter-laboratory comparability. Structural adjustments to study design are also needed to accelerate progress. Time-matched biospecimen sampling aligned with the elimination kinetics of target biomarkers is a requirement of the FoodBAll validation framework, yet remains the exception in practice. Controlled trials should expand beyond whole plant foods to include the processed plant-protein products that dominate the modern market. On the analytical side, hybrid workflows combining untargeted discovery with targeted validation represent the current best practice. The annotation bottleneck in untargeted metabolomics is being addressed by expanding spectral databases and developing harmonized data processing workflows with standard parameters. Multi-marker panels show considerable promise for capturing the complexity of plant-based dietary exposure, but require systematic external validation across diverse populations before routine epidemiological deployment. The combination of enhanced analytical capabilities, expanding food metabolomics databases and coordinated international validation efforts will drive substantial progress in the field. Achieving this is essential for objectively evaluating the health and sustainability outcomes of plant-based dietary patterns.
